# Supplementary material for: Vision, hearing, and intellectual disabilities in school-age children and adolescents (5–19 years) in Latin America and the Caribbean
Source: PLoS One. 2026 Sep 23;21(9):e0348693. doi: 10.1371/journal.pone.0348693 (PMC13600491; doi:10.1371/journal.pone.0348693)
Supplement: Supplementary file 1 [file pone.0348693.s001.pdf]

Table 1: **S1 Table.** Prevalence of Vision Loss by Country, Sex and Age Group - Latin America and Caribbean (5-19 years). Source: GBD 2023.

Country	Sex	Prev. 5-9 (%)	N Cases 5-9	Prev. 10-19 (%)	N Cases 10-19	Prev. 5-19 (%)	N Cases 5-19
Argentina	Male	0.87	13,081	1.27	43,246	1.01	56,327
	Female	1.19	18,042	1.49	52,102	1.29	70,144
	Both	1.03	31,123	1.38	95,349	1.15	126,471
Bahamas	Male	0.47	55	0.64	210	0.55	265
	Female	0.50	58	0.75	254	0.65	312
	Both	0.49	113	0.70	464	0.60	577
Barbados	Male	0.34	23	0.45	78	0.39	101
	Female	0.36	24	0.52	88	0.45	112
	Both	0.35	47	0.49	166	0.42	213
Belize	Male	0.46	91	0.65	262	0.56	353
	Female	0.50	93	0.76	306	0.65	399
	Both	0.48	184	0.70	568	0.60	752
Bolivia	Male	0.50	2,673	0.70	7,239	0.59	9,912
	Female	0.52	2,741	0.83	8,494	0.69	11,235
	Both	0.51	5,414	0.76	15,733	0.64	21,147
Brazil	Male	0.72	48,537	1.12	154,185	0.92	202,722
	Female	0.72	47,582	1.21	166,376	1.01	213,958
	Both	0.72	96,119	1.16	320,561	0.97	416,679
Chile	Male	0.80	4,323	1.14	13,735	0.91	18,058
	Female	1.10	5,894	1.36	16,270	1.18	22,164
	Both	0.95	10,217	1.25	30,005	1.04	40,222
Colombia	Male	0.53	8,636	0.67	24,406	0.55	33,042
	Female	0.60	10,056	0.99	36,419	0.82	46,476
	Both	0.56	18,693	0.83	60,825	0.68	79,518
Costa Rica	Male	0.47	750	0.61	2,216	0.51	2,965
	Female	0.55	926	0.90	3,328	0.74	4,253
	Both	0.51	1,675	0.75	5,544	0.63	7,219
Dominican Republic	Male	0.56	2,606	0.78	7,102	0.67	9,708
	Female	0.60	2,717	0.93	8,734	0.78	11,451
	Both	0.58	5,323	0.86	15,836	0.72	21,159
Ecuador	Male	0.43	2,991	0.59	9,162	0.51	12,153
	Female	0.45	3,069	0.70	10,714	0.60	13,783
	Both	0.44	6,060	0.65	19,876	0.55	25,936
El Salvador	Male	0.55	1,286	0.72	3,754	0.61	5,039
	Female	0.64	1,490	1.08	5,596	0.90	7,085
	Both	0.60	2,775	0.90	9,350	0.75	12,125
Guatemala	Male	0.59	4,799	0.77	12,052	0.65	16,851
	Female	0.68	5,643	1.16	18,719	0.96	24,362
	Both	0.63	10,442	0.96	30,772	0.80	41,214
Guyana	Male	0.48	165	0.66	438	0.56	603
	Female	0.51	172	0.77	516	0.65	688
	Both	0.49	337	0.71	954	0.60	1,291
Haiti	Male	0.49	3,597	0.67	9,018	0.59	12,615
	Female	0.52	3,726	0.80	10,595	0.68	14,321

Table 1 – continued from previous page

Country	Sex	Prev. 5-9 (%)	N Cases 5-9	Prev. 10-19 (%)	N Cases 10-19	Prev. 5-19 (%)	N Cases 5-19
	Both	0.50	7,323	0.74	19,613	0.63	26,936
Honduras	Male	0.46	2,564	0.60	6,343	0.52	8,907
	Female	0.56	3,053	0.94	9,936	0.78	12,989
	Both	0.51	5,617	0.77	16,279	0.65	21,896
Jamaica	Male	0.47	428	0.66	1,409	0.56	1,837
	Female	0.50	447	0.76	1,636	0.66	2,083
	Both	0.49	875	0.71	3,045	0.61	3,920
Mexico	Male	0.73	32,847	1.00	102,036	0.81	134,883
	Female	0.80	36,593	1.34	142,144	1.10	178,737
	Both	0.77	69,440	1.18	244,180	0.95	313,620
Panama	Male	0.52	842	0.66	2,167	0.55	3,009
	Female	0.58	954	0.93	3,097	0.77	4,052
	Both	0.55	1,796	0.79	5,264	0.66	7,060
Paraguay	Male	0.58	1,527	0.88	4,701	0.74	6,228
	Female	0.59	1,588	0.99	5,577	0.84	7,165
	Both	0.59	3,116	0.94	10,278	0.79	13,393
Peru	Male	0.54	8,097	0.78	23,374	0.68	31,471
	Female	0.58	8,461	0.96	28,121	0.82	36,582
	Both	0.56	16,558	0.87	51,495	0.75	68,053
Suriname	Male	0.52	123	0.72	346	0.61	469
	Female	0.55	123	0.83	382	0.70	505
	Both	0.53	245	0.77	728	0.66	973
Trinidad and Tobago	Male	0.52	215	0.72	653	0.61	868
	Female	0.56	221	0.82	749	0.70	970
	Both	0.54	436	0.77	1,402	0.65	1,838
Uruguay	Male	0.76	749	1.08	2,327	0.86	3,076
	Female	1.17	1,130	1.45	3,158	1.25	4,288
	Both	0.97	1,880	1.26	5,485	1.05	7,365
Venezuela	Male	0.44	4,031	0.57	11,723	0.50	15,754
	Female	0.53	4,789	0.88	18,511	0.75	23,299
	Both	0.48	8,820	0.72	30,234	0.62	39,054
LAC	Male	0.58	145,035	0.87	442,182	0.77	587,218
	Female	0.66	159,591	1.12	551,823	0.97	711,414
	Both	0.62	304,626	0.99	994,005	0.87	1,298,632
OECD Countries	Male	0.93	306,454	1.35	1,036,672	1.05	1,343,126
	Female	1.23	403,902	1.54	1,206,123	1.32	1,610,025
	Both	1.08	710,356	1.45	2,242,795	1.18	2,953,151
