# Supplementary material for: Vision, hearing, and intellectual disabilities in school-age children and adolescents (5–19 years) in Latin America and the Caribbean
Source: PLoS One. 2026 Sep 23;21(9):e0348693. doi: 10.1371/journal.pone.0348693 (PMC13600491; doi:10.1371/journal.pone.0348693)
Supplement: Supplementary file 2 [file pone.0348693.s002.pdf]

Table 1: **S2 Table.** Prevalence of Hearing Loss by Country, Sex and Age Group - Latin America and Caribbean (5-19 years). Source: GBD 2023.

Country	Sex	Prev. 5-9 (%)	N Cases 5-9	Prev. 10-19 (%)	N Cases 10-19	Prev. 5-19 (%)	N Cases 5-19
Argentina	Male	2.66	39,994	2.89	98,580	2.49	138,574
	Female	2.32	35,077	2.50	87,722	2.26	122,799
	Both	2.49	75,071	2.69	186,302	2.37	261,373
Bahamas	Male	3.61	424	4.46	1,462	3.94	1,887
	Female	3.08	354	3.66	1,236	3.33	1,590
	Both	3.35	779	4.05	2,698	3.64	3,477
Barbados	Male	3.57	243	4.46	768	3.90	1,011
	Female	3.00	197	3.67	618	3.30	816
	Both	3.29	440	4.07	1,386	3.60	1,826
Belize	Male	3.45	674	4.36	1,772	3.87	2,446
	Female	2.95	553	3.61	1,451	3.28	2,004
	Both	3.21	1,227	3.99	3,222	3.58	4,450
Bolivia	Male	3.27	17,656	4.18	43,444	3.61	61,100
	Female	2.79	14,598	3.47	35,669	3.11	50,267
	Both	3.04	32,254	3.82	79,113	3.36	111,367
Brazil	Male	4.04	271,256	5.09	703,002	4.43	974,258
	Female	3.40	223,901	4.15	571,659	3.77	795,560
	Both	3.72	495,157	4.62	1,274,661	4.10	1,769,818
Chile	Male	2.65	14,371	2.86	34,526	2.48	48,896
	Female	2.33	12,473	2.51	30,096	2.26	42,569
	Both	2.49	26,844	2.69	64,621	2.37	91,465
Colombia	Male	3.82	62,587	4.62	167,609	3.86	230,196
	Female	3.03	50,985	3.64	134,113	3.27	185,098
	Both	3.42	113,572	4.13	301,722	3.57	415,294
Costa Rica	Male	3.72	5,894	4.53	16,560	3.86	22,454
	Female	3.08	5,150	3.65	13,533	3.27	18,683
	Both	3.39	11,044	4.09	30,093	3.57	41,137
Dominican Republic	Male	3.59	16,597	4.42	40,095	3.88	56,692
	Female	3.06	13,941	3.65	34,427	3.29	48,368
	Both	3.33	30,538	4.03	74,522	3.59	105,060
Ecuador	Male	3.31	22,935	4.11	63,388	3.60	86,323
	Female	2.74	18,530	3.33	51,134	3.03	69,664
	Both	3.03	41,465	3.72	114,522	3.32	155,987
El Salvador	Male	3.69	8,551	4.54	23,627	3.87	32,178
	Female	3.00	6,945	3.63	18,815	3.26	25,760
	Both	3.34	15,496	4.09	42,442	3.57	57,938
Guatemala	Male	3.59	29,410	4.43	69,687	3.83	99,097
	Female	2.97	24,514	3.61	58,347	3.25	82,861
	Both	3.28	53,924	4.01	128,035	3.54	181,958
Guyana	Male	3.62	1,256	4.58	3,062	4.00	4,318
	Female	3.06	1,038	3.76	2,531	3.38	3,569
	Both	3.34	2,294	4.17	5,593	3.69	7,887
Haiti	Male	3.37	24,964	4.36	58,220	3.87	83,184
	Female	2.92	20,790	3.65	48,584	3.29	69,374

Table 1 – continued from previous page

Country	Sex	Prev. 5-9 (%)	N Cases 5-9	Prev. 10-19 (%)	N Cases 10-19	Prev. 5-19 (%)	N Cases 5-19
	Both	3.15	45,754	4.00	106,803	3.58	152,557
Honduras	Male	3.48	19,299	4.37	46,051	3.83	65,351
	Female	2.90	15,857	3.59	37,976	3.24	53,834
	Both	3.19	35,157	3.98	84,028	3.54	119,184
Jamaica	Male	3.52	3,176	4.45	9,538	3.89	12,714
	Female	2.96	2,644	3.63	7,785	3.30	10,429
	Both	3.24	5,819	4.04	17,324	3.60	23,143
Mexico	Male	4.00	178,697	5.02	510,197	4.14	688,894
	Female	3.24	147,881	3.93	416,998	3.48	564,879
	Both	3.62	326,579	4.46	927,195	3.82	1,253,774
Panama	Male	3.79	6,165	4.52	14,953	3.83	21,118
	Female	3.07	5,054	3.61	12,008	3.24	17,062
	Both	3.43	11,219	4.07	26,961	3.54	38,180
Paraguay	Male	3.88	10,213	4.74	25,377	4.21	35,590
	Female	3.22	8,656	3.86	21,742	3.55	30,399
	Both	3.54	18,869	4.29	47,119	3.88	65,989
Peru	Male	3.12	46,828	4.06	121,987	3.66	168,815
	Female	2.77	40,264	3.56	104,322	3.25	144,586
	Both	2.95	87,092	3.81	226,309	3.46	313,401
Suriname	Male	3.53	831	4.40	2,108	3.84	2,939
	Female	3.02	674	3.62	1,668	3.26	2,341
	Both	3.28	1,504	4.02	3,776	3.56	5,280
Trinidad and Tobago	Male	3.62	1,485	4.43	4,027	3.85	5,513
	Female	3.07	1,217	3.61	3,282	3.25	4,499
	Both	3.35	2,702	4.02	7,309	3.55	10,011
Uruguay	Male	2.69	2,646	2.89	6,237	2.47	8,883
	Female	2.35	2,266	2.50	5,456	2.25	7,722
	Both	2.52	4,912	2.69	11,693	2.36	16,605
Venezuela	Male	3.41	31,462	4.20	86,630	3.73	118,092
	Female	2.84	25,746	3.45	72,843	3.16	98,589
	Both	3.13	57,209	3.82	159,473	3.45	216,681
LAC	Male	3.28	817,615	4.23	2,152,906	3.92	2,970,522
	Female	2.82	679,305	3.59	1,774,017	3.34	2,453,322
	Both	3.05	1,496,921	3.92	3,926,923	3.63	5,423,844
OECD	Male	3.17	1,046,241	3.45	2,650,047	2.88	3,696,287
	Female	2.71	891,204	2.86	2,242,488	2.57	3,133,692
	Both	2.94	1,937,445	3.15	4,892,534	2.73	6,829,979
