# Supplementary material for: Vision, hearing, and intellectual disabilities in school-age children and adolescents (5–19 years) in Latin America and the Caribbean
Source: PLoS One. 2026 Sep 23;21(9):e0348693. doi: 10.1371/journal.pone.0348693 (PMC13600491; doi:10.1371/journal.pone.0348693)
Supplement: Supplementary file 3 [file pone.0348693.s003.pdf]

Table 1: **S3 Table.** Prevalence of Intellectual Developmental Disability by Country, Sex and Age Group - Latin America and Caribbean (5-19 years). Source: GBD 2023.

Country	Sex	Prev. 5-9 (%)	N Cases 5-9	Prev. 10-19 (%)	N Cases 10-19	Prev. 5-19 (%)	N Cases 5-19
Argentina	Both	2.32	70,054	2.09	144,899	1.95	214,953
	Male	2.83	42,507	2.56	87,505	2.33	130,012
	Female	1.82	27,547	1.64	57,394	1.56	84,941
Bahamas	Both	1.86	433	1.73	1,151	1.66	1,585
	Male	2.16	254	2.05	673	1.94	927
	Female	1.56	179	1.42	479	1.38	657
Barbados	Both	1.82	244	1.72	585	1.63	829
	Male	2.17	148	2.07	356	1.94	504
	Female	1.46	96	1.35	228	1.31	324
Belize	Both	1.83	699	1.76	1,421	1.70	2,120
	Male	2.10	410	2.07	842	1.98	1,252
	Female	1.54	289	1.44	579	1.42	868
Bolivia	Both	1.73	18,364	1.61	33,250	1.56	51,614
	Male	2.01	10,835	1.88	19,569	1.80	30,404
	Female	1.44	7,530	1.33	13,680	1.31	21,210
Brazil	Both	1.87	248,705	1.73	477,277	1.68	725,983
	Male	2.19	146,834	2.03	279,852	1.94	426,686
	Female	1.55	101,871	1.43	197,425	1.42	299,297
Chile	Both	2.26	24,304	2.03	48,728	1.89	73,033
	Male	2.76	14,950	2.48	29,952	2.27	44,902
	Female	1.75	9,355	1.57	18,776	1.50	28,131
Colombia	Both	1.93	64,036	1.72	125,830	1.63	189,866
	Male	2.34	38,298	2.07	74,999	1.90	113,297
	Female	1.53	25,738	1.38	50,830	1.35	76,568
Costa Rica	Both	1.79	5,831	1.62	11,955	1.54	17,786
	Male	2.15	3,409	1.95	7,104	1.81	10,513
	Female	1.45	2,422	1.31	4,852	1.27	7,274
Dominican Republic	Both	1.86	17,062	1.76	32,589	1.69	49,651
	Male	2.15	9,920	2.08	18,868	1.97	28,788
	Female	1.57	7,142	1.46	13,721	1.42	20,864
Ecuador	Both	1.75	23,907	1.63	50,126	1.58	74,033
	Male	2.03	14,063	1.90	29,345	1.81	43,407
	Female	1.46	9,845	1.35	20,781	1.33	30,626
El Salvador	Both	1.94	9,000	1.75	18,171	1.68	27,172
	Male	2.31	5,350	2.07	10,793	1.94	16,143
	Female	1.58	3,650	1.42	7,378	1.40	11,028
Guatemala	Both	2.08	34,163	1.86	59,482	1.82	93,645
	Male	2.43	19,939	2.19	34,389	2.10	54,328
	Female	1.72	14,224	1.55	25,094	1.54	39,318
Guyana	Both	1.99	1,367	1.89	2,542	1.83	3,910
	Male	2.29	794	2.18	1,460	2.09	2,254
	Female	1.69	573	1.61	1,083	1.57	1,656
Haiti	Both	2.00	29,069	1.87	49,970	1.86	79,038

Table 1 – continued from previous page

Country	Sex	Prev. 5-9 (%)	N Cases 5-9	Prev. 10-19 (%)	N Cases 10-19	Prev. 5-19 (%)	N Cases 5-19
	Male	2.26	16,694	2.14	28,563	2.11	45,257
	Female	1.74	12,374	1.61	21,407	1.60	33,781
Honduras	Both	2.00	22,000	1.83	38,556	1.80	60,556
	Male	2.32	12,844	2.12	22,300	2.06	35,144
	Female	1.67	9,156	1.54	16,256	1.53	25,412
Jamaica	Both	1.91	3,422	1.82	7,797	1.74	11,219
	Male	2.20	1,989	2.14	4,581	2.01	6,570
	Female	1.61	1,433	1.50	3,216	1.47	4,650
Mexico	Both	2.08	187,530	1.86	386,779	1.75	574,309
	Male	2.52	112,461	2.27	230,628	2.06	343,089
	Female	1.65	75,069	1.47	156,151	1.42	231,220
Panama	Both	1.82	5,956	1.63	10,795	1.55	16,751
	Male	2.18	3,549	1.94	6,425	1.81	9,973
	Female	1.46	2,408	1.31	4,370	1.29	6,778
Paraguay	Both	1.75	9,291	1.63	17,891	1.60	27,183
	Male	2.05	5,392	1.92	10,263	1.85	15,655
	Female	1.45	3,900	1.35	7,628	1.35	11,528
Peru	Both	1.62	47,964	1.54	91,250	1.53	139,214
	Male	1.89	28,357	1.79	53,793	1.78	82,150
	Female	1.35	19,607	1.28	37,457	1.28	57,064
Suriname	Both	1.94	891	1.80	1,697	1.75	2,588
	Male	2.25	530	2.12	1,017	2.02	1,546
	Female	1.62	362	1.48	680	1.45	1,042
Trinidad and Tobago	Both	2.01	1,620	2.02	3,672	1.88	5,292
	Male	2.27	934	2.32	2,113	2.13	3,048
	Female	1.73	685	1.71	1,559	1.62	2,244
Uruguay	Both	2.30	4,478	2.03	8,825	1.89	13,302
	Male	2.80	2,749	2.49	5,375	2.26	8,124
	Female	1.79	1,729	1.58	3,450	1.51	5,178
Venezuela	Both	1.86	33,926	1.70	70,987	1.67	104,912
	Male	2.22	20,461	2.04	41,971	1.97	62,431
	Female	1.49	13,465	1.37	29,016	1.36	42,481
LAC	Both	1.76	864,318	1.69	1,696,227	1.71	2,560,545
	Male	2.06	513,670	1.97	1,002,735	2.00	1,516,405
	Female	1.46	350,648	1.40	693,492	1.42	1,044,140
OECD	Both	2.50	1,645,258	2.13	3,310,898	1.98	4,956,156
	Male	3.08	1,015,728	2.66	2,042,567	2.39	3,058,294
	Female	1.92	629,531	1.62	1,268,331	1.55	1,897,862
