# Supplementary material for: Vision, hearing, and intellectual disabilities in school-age children and adolescents (5–19 years) in Latin America and the Caribbean
Source: PLoS One. 2026 Sep 23;21(9):e0348693. doi: 10.1371/journal.pone.0348693 (PMC13600491; doi:10.1371/journal.pone.0348693)
Supplement: Supplementary file 4 [file pone.0348693.s004.pdf]

Table 1: **S4 Table.** Visual Severity Distribution by Country - LAC (Percentages). Source: GBD 2023.

Country	Blindness	Severe vision loss	Moderate vision loss	Presbyopia	Total
Argentina	4.22%	6.91%	87.96%	0.91%	100.0%
Bahamas	4.43%	5.38%	84.52%	5.66%	100.0%
Barbados	2.95%	6.79%	82.12%	8.14%	100.0%
Belize	5.20%	5.51%	83.53%	5.76%	100.0%
Bolivia	6.64%	6.29%	82.65%	4.42%	100.0%
Brazil	4.47%	5.05%	88.15%	2.33%	100.0%
Chile	4.49%	5.92%	88.59%	1.00%	100.0%
Colombia	4.85%	6.39%	84.24%	4.53%	100.0%
Costa Rica	5.17%	5.53%	84.38%	4.93%	100.0%
Dominican Republic	5.23%	4.93%	85.09%	4.75%	100.0%
Ecuador	4.78%	6.26%	83.79%	5.17%	100.0%
El Salvador	5.34%	6.37%	84.05%	4.24%	100.0%
Guatemala	6.00%	6.88%	83.20%	3.93%	100.0%
Guyana	5.66%	5.49%	83.32%	5.53%	100.0%
Haiti	7.76%	5.16%	81.47%	5.61%	100.0%
Honduras	5.27%	5.56%	84.35%	4.82%	100.0%
Jamaica	4.73%	5.57%	83.98%	5.72%	100.0%
Mexico	3.99%	6.66%	86.52%	2.83%	100.0%
Panama	6.27%	6.74%	82.37%	4.62%	100.0%
Paraguay	4.56%	5.79%	86.05%	3.60%	100.0%
Peru	6.29%	6.27%	83.66%	3.78%	100.0%
Suriname	7.39%	5.21%	82.21%	5.19%	100.0%
Trinidad and Tobago	4.96%	5.01%	81.80%	8.24%	100.0%
Uruguay	4.34%	5.26%	89.40%	1.00%	100.0%
Venezuela	6.46%	6.20%	82.18%	5.16%	100.0%
LAC	4.72%	5.97%	86.37%	2.94%	100.0%
OECD Countries	4.27%	5.81%	88.58%	1.34%	100.0%
