# Supplementary material for: Vision, hearing, and intellectual disabilities in school-age children and adolescents (5–19 years) in Latin America and the Caribbean
Source: PLoS One. 2026 Sep 23;21(9):e0348693. doi: 10.1371/journal.pone.0348693 (PMC13600491; doi:10.1371/journal.pone.0348693)
Supplement: Supplementary file 5 [file pone.0348693.s005.pdf]

Table 1: **S5 Table.** Hearing Loss Severity Distribution by Country - GBD Classification (%) - LAC 5-19 years. Source: GBD 2023.

Country	Complete ($\geq$95 dB)	Profound (80-94 dB)	Severe (65-79 dB)	Mod. Severe (50-64 dB)	Moderate (35-49 dB)	Mild (20-34 dB)	Total
Argentina	0.86%	3.58%	2.42%	4.94%	21.65%	66.55%	100.0%
Bahamas	0.31%	5.20%	3.45%	3.71%	26.74%	60.60%	100.0%
Barbados	0.33%	5.20%	3.20%	3.85%	25.91%	61.51%	100.0%
Belize	0.39%	5.16%	3.71%	3.58%	26.38%	60.77%	100.0%
Bolivia	0.53%	6.59%	4.21%	4.51%	20.22%	63.94%	100.0%
Brazil	0.23%	4.15%	3.17%	3.15%	34.05%	55.25%	100.0%
Chile	0.80%	3.58%	3.22%	4.61%	22.56%	65.23%	100.0%
Colombia	0.36%	5.21%	2.84%	4.08%	24.83%	62.68%	100.0%
Costa Rica	0.35%	5.20%	2.81%	4.08%	24.88%	62.68%	100.0%
Dominican Republic	0.38%	5.13%	3.70%	3.55%	26.77%	60.47%	100.0%
Ecuador	0.52%	6.15%	3.97%	4.31%	20.14%	64.90%	100.0%
El Salvador	0.42%	5.26%	3.05%	4.03%	24.91%	62.32%	100.0%
Guatemala	0.44%	5.22%	3.09%	3.98%	24.91%	62.35%	100.0%
Guyana	0.34%	4.92%	3.49%	3.44%	26.43%	61.39%	100.0%
Haiti	0.50%	5.13%	3.89%	3.47%	25.66%	61.34%	100.0%
Honduras	0.46%	5.23%	3.15%	3.97%	24.65%	62.54%	100.0%
Jamaica	0.36%	5.20%	3.61%	3.65%	26.26%	60.93%	100.0%
Mexico	0.39%	5.28%	3.43%	4.04%	27.29%	59.57%	100.0%
Panama	0.35%	5.19%	2.83%	4.09%	25.09%	62.45%	100.0%
Paraguay	0.21%	4.15%	2.98%	2.81%	32.83%	57.03%	100.0%
Peru	0.44%	7.05%	4.21%	4.87%	21.08%	62.34%	100.0%
Suriname	0.38%	5.19%	3.74%	3.60%	26.65%	60.44%	100.0%
Trinidad and Tobago	0.33%	5.20%	3.60%	3.66%	26.81%	60.40%	100.0%
Uruguay	0.87%	3.59%	2.39%	4.96%	21.62%	66.57%	100.0%
Venezuela	0.44%	5.48%	3.16%	4.20%	24.87%	61.84%	100.0%
LAC TOTAL	0.38%	4.93%	3.29%	3.83%	27.94%	59.63%	100.0%
OECD COUNTRIES	0.82%	4.26%	2.52%	4.63%	23.00%	64.76%	100.0%
