# Supplementary material for: Vision, hearing, and intellectual disabilities in school-age children and adolescents (5–19 years) in Latin America and the Caribbean
Source: PLoS One. 2026 Sep 23;21(9):e0348693. doi: 10.1371/journal.pone.0348693 (PMC13600491; doi:10.1371/journal.pone.0348693)
Supplement: Supplementary file 6 [file pone.0348693.s006.pdf]

Table 1: **S6 Table.** Severity Distribution of Intellectual Developmental Disability - LAC (5-19 years). Source: GBD 2023.

Country	Borderline	Mild	Moderate	Severe	Profound	Total
LAC	13.38%	48.31%	18.49%	15.80%	4.02%	100
Argentina	15.81%	46.70%	18.64%	14.82%	4.04%	100
Bahamas	12.09%	46.90%	19.52%	17.65%	3.84%	100
Barbados	12.64%	47.11%	19.20%	17.17%	3.88%	100
Belize	11.34%	47.87%	19.09%	17.84%	3.86%	100
Bolivia	15.68%	49.84%	16.92%	13.26%	4.29%	100
Brazil	11.33%	50.51%	17.97%	16.15%	4.04%	100
Chile	16.47%	45.73%	19.23%	14.52%	4.05%	100
Colombia	13.90%	46.30%	19.40%	16.51%	3.88%	100
Costa Rica	14.88%	46.85%	18.94%	15.34%	3.99%	100
Dominican Republic	10.81%	46.35%	20.01%	19.10%	3.71%	100
Ecuador	13.57%	51.00%	17.11%	14.09%	4.23%	100
El Salvador	13.58%	47.92%	18.70%	15.79%	4.00%	100
Guatemala	12.85%	47.47%	19.12%	16.63%	3.93%	100
Guyana	10.27%	45.89%	20.36%	19.82%	3.66%	100
Haiti	13.20%	49.33%	17.86%	15.54%	4.07%	100
Honduras	13.03%	46.28%	19.48%	17.33%	3.88%	100
Jamaica	11.34%	45.26%	20.37%	19.36%	3.67%	100
Mexico	14.36%	46.86%	18.91%	15.88%	3.99%	100
Panama	14.50%	47.29%	18.84%	15.37%	4.01%	100
Paraguay	11.86%	48.97%	18.51%	16.68%	3.97%	100
Peru	13.45%	50.44%	17.52%	14.42%	4.17%	100
Suriname	11.40%	47.56%	19.20%	18.01%	3.83%	100
Trinidad and Tobago	10.45%	41.92%	22.30%	21.93%	3.40%	100
Uruguay	16.82%	48.05%	17.94%	12.97%	4.22%	100
Venezuela	14.82%	47.46%	18.46%	15.22%	4.04%	100
OECD Countries	16.93%	47.16%	18.48%	13.30%	4.14%	100
