# Supplementary material for: Vision, hearing, and intellectual disabilities in school-age children and adolescents (5–19 years) in Latin America and the Caribbean
Source: PLoS One. 2026 Sep 23;21(9):e0348693. doi: 10.1371/journal.pone.0348693 (PMC13600491; doi:10.1371/journal.pone.0348693)
Supplement: Supplementary file 7 [file pone.0348693.s007.pdf]

Table 1: **S7 Table.** Return on investment (ROI) assumptions and input parameters by condition. Source: study economic model (see Methods, Economic Analysis).

Condition	Intervention	Cost Inputs	Benefit Components	Horizon / Discount
**Vision**	School-based screening + spectacles	US$35 per child (US$5-15 screening + US$15-35 glasses)	US$320 annual productivity gain (0.4 extra schooling years × 10% wage return × US$8,000 baseline); PV = US$7,395	10 years (3%); lifetime sensitivity provided
**Hearing**	Universal newborn hearing screening + treatment (hearing aids, cochlear implants, follow-up)	US$10.5-12.8B initial (5 yrs) + US$1.41-1.92B annual recurring	US$129.6B productivity + US$48.6B education savings + US$8-12B healthcare savings (30 yrs, 60% treatment uptake)	30 years (3%); break-even 8-11 yrs
**Intellectual**	Early intervention (0-3 yrs) – clinic/community models	US$8,000-25,000 per child over 3 years	US$18-36k education savings + US$40-80k productivity + US$200-400k residential care avoided + US$5-15k health savings	20 years (3%); break-even 4-8 yrs

Data Sources:

- Vision: IDB policy brief on vision loss and blindness in school-age children in LAC (2025)
- Hearing: IDB policy brief on hearing loss and deafness in school-age children in LAC (2025)
- Intellectual: IDB analytical inputs on intellectual disability in school-age children in LAC (forthcoming)

Notes: All ROI calculations use a 3% annual discount rate following WHO-CHOICE guidelines. Sensitivity analyses varying cost inputs, effectiveness parameters, and discount rates confirm robustness of cost-effectiveness conclusions across scenarios.
